# Copeptin: a neuroendocrine biomarker of COVID-19 severity

**DOI:** 10.2217/bmm-2021-1100

**Published:** 2022-03-30

**Authors:** Reham Hammad, Ahmed Elshafei, Emad Gamil Khidr, Ahmed A El-Husseiny, Maher H Gomaa, Hend G Kotb, Heba H Eltrawy, Hesham Farhoud

**Affiliations:** ^1^Department of Clinical Pathology, Faculty of Medicine (Girls), Al-Azhar University, Cairo, 11884, Egypt; ^2^Biochemistry & Molecular Biology Department, Faculty of Pharmacy (Boys), Al-Azhar University, Cairo, 11884, Egypt; ^3^Internal Medicine Department, Faculty of Medicine (Girls), Al-Azhar University, Cairo, 11884, Egypt; ^4^Chest Diseases Department, Faculty of Medicine (Girls), Al-Azhar University, Cairo, 11884, Egypt; ^5^Orthopedic Surgery Department, Dean of Faculty of Medicine (Girls), Al-Azhar University, Cairo, 11884, Egypt

**Keywords:** arginine vasopressin, copeptin, coronavirus, COVID-19, stress

## Abstract

**Aim:** To investigate the change in a serum level of copeptin, a neuroendocrine biomarker, in differentiating grades of COVID-19 severity on admission time and to find its diagnostic potential. **Materials & Methods:** 160 COVID-19 patients were classified according to disease severity into 80 mild to moderate and 80 severe patients. Serum copeptin level was assessed by ELISA on their admission time. Besides, serum CRP, ferritin and D-dimer were estimated. **Results:** Severe COVID-19 patients showed higher serum copeptin level in comparison to mild to moderate cases, with diagnostic potential to distinguish disease severity with 93.33% sensitivity and 100% specificity at cut-off value >18.5 Pmol/l. **Conclusion:** Serum copeptin was remarkably increased with COVID-19 severity with reasonable differentiation potential for recently admitted patients.

## Plain language summary

We conducted a biochemical study on the role of copeptin – a biomarker of acute stress due to COVID-19 infection – in classification of COVID-19 severity on admission over 160 adult patients. Copeptin was highly elevated in severe cases more than the mild to moderate ones. So, it may be an early marker in admission departments to ease early clinical decisions and medical intervention.

COVID-19 is a rapid-spreading infectious disease that emerged in Wuhan, China at the end of 2019 and was described as a global pandemic health problem by the world health organization with a remarkable impact on mortality rates and health care resources [1,2]. The causative virus was recognized as SARS-CoV-2 that is a member of the coronavirus β-family [3]. Infection of humans with SARS-CoV-2 can result in mild to severe respiratory illness [4].

Interestingly, most COVID-19 cases have mild symptoms at the beginning of infection. However, some cases worsen rapidly to severe symptoms like metabolic acidosis, dyspnea, sepsis and acute respiratory distress syndrome (ARDS) and even death [5]. Finding out a new biochemical marker for evaluating the severity of COVID-19 can facilitate suitable supportive care, symptoms management and consequently decreases mortality.

Stress, inflammation and pain are the consequences of COVID-19 infection. Stress stimulates arginine vasopressin (AVP) secretion through the activation of the hypothalamic-hypophyseal axis or cortical neurons induced hypothalamus [6,7]. Besides, lung injury may lead to hypoxic pulmonary vasoconstriction causing an insufficient filling of the left atrium with a subsequent increase of AVP [8]. Moreover, IL-6 and other inflammatory cytokines can activate the non-osmotic secretion of AVP [9,10].

Arginine vasopressin, identified as an antidiuretic hormone, is synthesized as a preprotein comprising AVP, neurophysin 2 and copeptin in paraventricular and supraoptic nuclei of the hypothalamus [11,12]. It promotes stress conditioning, vasoconstriction and blood volume regulation. Besides, its secretion is raised in response to hyperosmolarity, reduced blood pressure, hypoxia, hypovolemia, infections and respiratory or metabolic acidosis [13,14].

Copeptin is a glycopeptide of 39 amino acids originating from the C-terminus of the AVP preprotein. Together with AVP, copeptin is secreted in an equimolar amount from the neurohypophysis in response to AVP release stimulating conditions [15]. Due to the short half-life and instability of AVP in blood, copeptin is a preferred sensitive and stable surrogate biomarker for estimating AVP release [16,17].

This study is aimed to find the impact of COVID-19 severity on the serum copeptin level as a stress biomarker for the infection consequences. Additional objectives are to correlate this impact with the other inflammatory biomarkers of COVID-19 such as C-reactive protein (CRP), ferritin and D-dimer and explore its potential as a differentiating tool between the grades of COVID-19 severity.

## Materials & methods

### Study participants

This prospective study was conducted on 160 PCR confirmed COVID-19 patients. They were recruited from Al-Zahraa Hospital, Faculty of Medicine (Girls), Al-Azhar University, Cairo, Egypt named for SARS-CoV-2 positive patients' isolation. Patients were classified according to disease severity based on the guidance of the Egyptian ministry of health and population (MOHP) [18] into 80 mild to moderate COVID-19 patients and 80 severe COVID-19 patients. Mild to moderate cases met the following criteria: fever and respiratory symptoms, CORAD 1–5 and oxygen saturation (SpO_2_ ≥92%) while severe cases met the following criteria: fever and respiratory symptoms, CORAD 4–5 and oxygen saturation (SpO_2_ <92%). A free informed consent form was signed by all participants or their corresponding companions. The study was done after approval of the research ethics committee of Faculty of Medicine (Girls), Al-Azhar University, Cairo, Egypt with approval number (202106884) and was in accordance with the tenets of the Helsinki Declaration. Moreover, this study protocol was registered in ClinicalTrial.gov database with identifier ID (NCT05249751). Exclusion criteria included pregnancy, COVID-19 patients with morbid obesity, malignancy, autoimmune diseases and those who received immunomodulators or began COVID-19 treatment protocol. Also, patients with a history of recurrent COVID-19 were excluded.

### Sampling & methodology

A total of 8 ml of venous blood was taken from each subject upon admission. Blood sample was divided into three tubes. The first tube was containing EDTA in which 2 ml of blood was transferred for performing complete blood count (Sysmex KX-21, Japan) and flow cytometry assay. The second tube was for serum separating in which 4 ml was centrifuged. Serum was divided into four parts. The first portion was used for measurement of creatinine, ALT and ferritin spectrophotometrically (Cobas Integra 400 plus, Roche diagnostics, Germany). The second portion was used for the measurement of sodium (Na^+^) and potassium (K^+^), by electrolyte analyzer (AVL 9180, Roche diagnostics, Germany). The third portion was used for measurement of CRP turbidimetrically (Cobas Integra 400 plus, Roche diagnostics, Germany). The fourth portion of serum was used for measurement of copeptin levels using its commercially available ELISA technique-based kits supplied by Phoenix Pharmaceuticals, Inc, USA (Stat Fax^®^ 2100 Microplate Reader, Awareness Technology, USA). The third tube was containing sodium citrate in which the last 2 ml of blood was transferred for measuring D-dimer by immunoassay technique (Cobas h 232, Roche diagnostics, Germany).

Flow cytometry was conducted for the estimation of T lymphocytes percentage (T cells %) using four-color FACS Calibur (BD Biosciences, CA, USA). Cell Quest Pro software (BD Biosciences) was used for data analysis. The compensation setting was established before acquiring the samples using color-calibrated beads.

### Statistical analysis

Data were collected and analyzed using GraphPad Prism 9 (GraphPad Software, CA, USA). Quantitative data were expressed as mean ± standard error (SE). Qualitative data were expressed as frequency and percentage.

All quantitative data were checked for normality using D'Agostino–Pearson Omnibus test. Mann–Whitney test was used for comparing quantitative data while Chi square (X^2^) was used for comparison between categorical data. Spearman's rank correlation coefficient was used for assessing the relationship between serum copeptin and other variables. Receiver operating characteristic (ROC) curves were generated to find the differentiating power of copeptin, CRP, ferritin and D-dimer between COVID-19 cases severity. Multiple logistic regression analysis was performed for the detection of predictors for COVID-19 severity. p-value <0.05 was considered as statistically significant.

## Results

The study population consisted of 160 COVID-19 patients (80 mild to moderate cases and 80 severe cases). The demographical, clinical and biochemical data of the two groups are presented in Table 1. No statistically significant difference was determined among the two groups in terms of sex while the ages of patients in severe cases were significantly higher compared to mild to moderate cases.

**Table 1.** Demographic, clinical and biochemical data of the studied groups.

Studiedgroup variable	Mild to moderate COVID-19 patients (n = 80)	Severe COVID-19 patients (n = 80)	p-value
Gender (male/female)	39 (48.7)/41 (51.3)	40 (50)/40 (50)	0.874
Age (years)	44.7 ± 2.92	62.5 ± 2.21	<0.001^†^
Oxygen saturation (%)	96.4 ± 0.24	82.9 ± 1.65	<0.001^†^
Generalized bone ache	36 (45)	80 (100)	<0.001^†^
Fever	43 (53.7)	80 (100)	<0.001^†^
Cough	45 (56.2)	80 (100)	<0.001^†^
Dyspnea	28 (35)	80 (100)	<0.001^†^
GIT manifestations	23 (28.7)	33 (41.2)	0.97
Diabetes mellitus	12 (15)	44 (55)	<0.001^†^
Hypertension	14 (17.5)	46 (57.5)	<0.001^†^
Bronchial asthma	13 (16.2)	6 (7.5)	0.087
TLC (X 10^3^/mm^3^)	7.41 ± 0.6	9.92 ± 1.1	0.0514
ANC (X 10^3^/mm^3^)	5 ± 0.49	8.02 ± 1	0.007^†^
Hemoglobin (g/dl)	11.9 ± 0.37	10.9 ± 0.35	0.066
PLT (X 10^3^/mm^3^)	241 ± 19.5	247 ± 25	0.849
T cells (%)	61.1 ± 1.72	37.3 ± 3.62	<0.001^†^
CRP (mg/l)	5.31 ± 2.72	30.4 ± 7.27	<0.001^†^
Ferritin (ng/ml)	85.6 ± 31.4	411 ± 76.1	<0.001^†^
D-dimer (ng/ml)	405 ± 49.4	1081 ± 196	<0.001^†^
ALT (U/l)	20.1 ± 2.21	26.4 ± 3.14	0.056
Creatinine (mg/dl)	0.81 ± 0.3	1.27 ± 0.3	0.507
Na^+^ (mmol/l)	141 ± 0.97	139 ± 1.02	<0.001^†^
K^+^ (mmol/l)	4.09 ± 0.06	3.62 ± 0.09	<0.001^†^
Copeptin (Pmol/l)	13.7 ± 0.61	30.1 ± 1.96	<0.001^†^

† Significant at p < 0.05.

Data are expressed as mean ± SE or n (%).

ANC: Absolute neutrophil count; ALT: Alanine transaminase; CRP: C-reactive protein; CT: Computed tomography; GIT: Gastrointestinal tract; PLT: Platelet count; TLC: Total leukocyte count.

Severe COVID-19 patients showed a significantly higher absolute neutrophil count (ANC), CRP, ferritin and D-dimer levels in addition to significantly lower T cells %, blood sodium, and potassium levels than patients with mild to moderate COVID-19.

The mean serum copeptin levels of mild to moderate and severe COVID-19 patients were 13.7 ± 0.61 and 30.1 ± 1.96 Pmol/l, respectively. Serum copeptin levels were significantly higher in the severe COVID-19 group compared to the mild to moderate group according to these results (p < 0.001).

The correlation findings between serum copeptin and other parameters are enlisted in Table 2. Copeptin showed mild to moderate strength linear positive correlation with CRP, ferritin and D-dimer (r = 0.658, p < 0.0001; r = 0.636, p < 0.0001; r = 0.474, p < 0.0001, respectively). While there were moderate-strong linear negative correlations between copeptin and O_2_ saturation %, T cells %, Na^+^ and K^+^ levels (r = −0.714, p < 0.0001; r = −0.588, p < 0.0001; r = −0.51, p < 0.0001; r = −0.44, p = 0.0005, respectively).

**Table 2.** Correlation between serum copeptin and hematological and biochemical parameters of the studied groups.

Clinical & Laboratory Variables	Serum Copeptin	
	R	p-value
O_2_ saturation (%)	-0.714	<0.0001^†^
TLC (X 10^3^/mm^3^)	0.137	0.28
ANC (X 10^3^/mm^3^)	0.167	0.207
Hemoglobin (g/dl)	-0.237	0.069
PLT (X 10^3^/mm^3^)	-0.074	0.573
T cells (%)	-0.588	<0.0001^†^
CRP (mg/l)	0.658	<0.0001^†^
Ferritin (ng/ml)	0.636	<0.0001^†^
D-dimer (ng/ml)	0.474	<0.0001^†^
Na^+^ (mmol/l)	-0.51	<0.0001^†^
K^+^ (mmol/l)	-0.44	0.0005^†^

† Significant at p < 0.05.

ANC: Absolute neutrophil count; CRP: C-reactive protein; PLT: Platelet count; TLC: Total leukocyte count.

**Figure 1.**
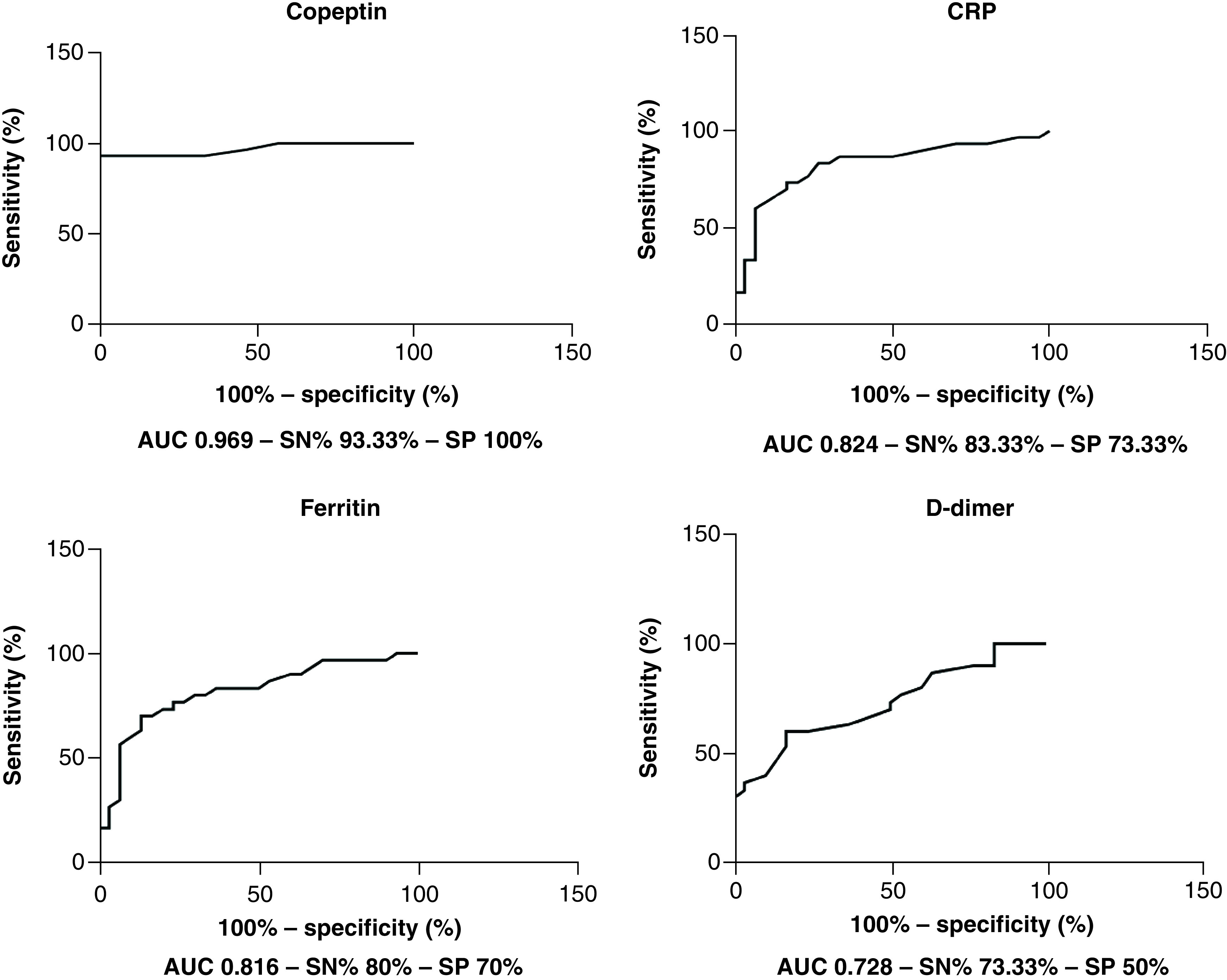
Receiver operating characteristic curves for copeptin, CRP, Ferritin and D-dimer as biomarkers of severity in COVID-19 patients. AUC: Area under the curve; CRP: C-reactive protein; SN: Sensitivity; SP: Specificity.

To find the most reliable biomarker to distinguish severe COVID-19 cases, the ROC curves for CRP, ferritin, D-dimer and copeptin were performed (Figure 1 & Table 3). Results revealed that copeptin had the most diagnostic potential to differentiate severe COVID-19 cases from mild to moderate ones with a sensitivity 93.33% and specificity 100% at cut-off value >18.5 Pmol/l.

**Table 3.** Receiver operating characteristic curves data for CRP, ferritin, D-dimer and copeptin as biomarkers of severity in COVID-19 Patients.

Biomarker	Cut-off point	AUC	Sensitivity (%)	Specificity (%)	95% CI	p-value
CRP	>3.55 mg/l	0.824	83.33	73.33	0.713–0.935	<0.0001^†^
Ferritin	>57.15 ng/ml	0.816	80	70	0.705–0.926	<0.0001^†^
D-dimer	>421 ng/ml	0.728	73.33	50	0.600–0.855	0.002^†^
Copeptin	>18.50 Pmol/l	0.969	93.33	100	0.926–1.013	<0.0001^†^

† Significant at p < 0.05.

AUC: Area under the curve; CRP: C-reactive protein.

Furthermore, multiple logistic regression analysis of age, diabetes mellitus and hypertension, copeptin, CRP, ferritin and D-dimer with COVID-19 severity found that copeptin was an independent predictor for COVID-19 severity (OR = 2.001, 95% CI: 1.388–3.596, p = 0.003) as shown in Table 4.

**Table 4.** Multiple regression analysis of predictors for COVID-19 severity.

Variables	p-value	OR	95% CI for OR	
			Lower limit	Upper limit
Copeptin	0.003^†^	2.001	1.388	3.596
CRP	0.589	1.036	0.916	1.177
Ferritin	0.332	0.994	0.986	1.004
D-dimer	0.942	1.000	0.997	1.005

† Significant at p < 0.05.

CRP: C-reactive protein; OR: Odds ratio.

## Discussion

Endocrine stress conditioning is mediated by many hormones, including the AVP and cortisol through the activation of the hypothalamic–adenohypophyseal axis. Copeptin is a biochemical mirror to the AVP as being synthesized from the same ancestor gene, preproprotein precursor and stoichiometrically released, in addition to being remarkably stable in blood samples with the easier biochemical assay [16,17].

The inflammation, pain, lung injury, plasma osmolarity changes and psychological stress associated with COVID-19 are behind the activation of the stress-adapting endocrine axis with a consequent increase in circulating AVP/copeptin [6,7]. Therefore, we aimed in this study to investigate the changes in serum copeptin level, and its differentiating power of COVID-19 cases severity on admission time.

This study revealed a significant increase in serum copeptin level in severe COVID-19 patients in comparison to the mild to moderate cases. Our findings were in accord with previous studies on COVID-19 patients [13], and other respiratory infections like pneumonia [14], where the circulating copeptin level was raised and correlated with the severity and poor prognosis [15,19].

Additionally, the differentiating power of copeptin between the COVID-19 patients' severity was nearly similar to the finding of *Gregoriano* *et al.*, where their optimal cut-off value was 20 Pmol/l with a sensitivity 88.2% and specificity 64.9% [13], while our optimal cut-off value was 18.5 Pmol/l with sensitivity 93.33% and specificity 100%. This accordance with the results would augment the potentiality of copeptin as a biomarker for COVID-19 severity stratification.

The pathophysiological mechanisms behind the occurrence of the syndrome of inappropriate antidiuretic hormone secretion in pneumonia as in COVID-19 are not fully elucidated but may be due to a non-osmolar release of AVP secondary to intravascular fluid depletion, activating the baroreceptors, which in turn activates the rennin-angiotensin-aldosterone system [19].

SARS-CoV-2-infected person is usually suffering from accompanying emotional, psychological, physical changes in addition to stress and pain [19]. These changes can stimulate AVP/copeptin release through the hypothalamic–neurohypophyseal pathway. Additionally, stress can induce direct AVP/corticotropin-releasing hormone (CRH) release through activation of the hypothalamic–adenohypophyseal pathway [20–22].

Moreover, SARS-CoV-2 respiratory infection resembles other infectious conditions and sepsis, where AVP/copeptin is released as a stress-adapting and homeostatic hormone to maintain the euvolemic state, blood pressure with persevered renal functions [15,23,24].

The COVID-19-induced lung injury may affect the cardiopulmonary hemodynamics and left atrial underfilling with subsequent activation of renin–angiotensin–aldosterone system and AVP/copeptin release. This could happen from either the direct viral-induced injury or the cytokines storm-induced injury [19,25–27].

In a harmony with previous studies [28–30], our study reported highly significant CRP levels in severe cases as compared to mild to moderate ones.

In a line with a meta-analysis performed by Taneri *et al.*, severe COVID-19 patients have significantly higher levels of ferritin than mild to moderate cases [31]. Kernan and Carcillo suggested a feedback system between ferritin and cytokines, in which cytokines can stimulate ferritin expression and ferritin can induce pro-and anti-inflammatory cytokine expression [32].

In accordance with many reports [5,33,34], the levels of D-dimer in severe patients were significantly higher than in mild to moderate ones. The upregulation of tissue factors in sepsis leads to the downregulation of anti-thrombin. As a result, the level of plasma thrombin rises. Fibrinolysis is further inhibited by a decrease in protein C synthesis and an increase in type 1 plasminogen activator inhibitor. All these changes have the potential to cause hypercoagulability with a consequent increase in D-dimer levels as an indicator of active fibrinolysis [35].

Significant hyponatremia and hypokalemia were observed in severe cases which may be a result of activation of the vasopressinergic system with the progression of disease severity. These observations are consistent with earlier reports [36–38]. The proximal tubule is one of the ACE2 receptor's expression sites [39], and enhanced ACE2 receptor expression in the proximal tubule can cause hyponatremia and hypokalemia [40].

Severe COVID-19 cases were associated with a remarkable rise in ANC in agreement with several studies [41,42]. The COVID-19 responsive inflammatory molecules, such as IL-6 and IL-8 and tumor necrosis factor-α may be beyond these changes [43]. Moreover, circulating T lymphocytes in severe cases showed a significant decrease as was found in other studies [41,44].

Interestingly, our study revealed that serum copeptin positively correlated with the inflammatory markers; CRP, ferritin, and D-dimer, while showed a negative correlation with T-cells%. These results could be explained as the severity of COVID-19 is associated with cytokines storm [45], an especially dramatic increase in IL-6, which in turn could activate the hypothalamic-adenohypophyseal axis to release AVP/copeptin and CRH [26].

On the other hand, serum sodium level was negatively correlated with copeptin. This finding could support the role of the vasopressinergic system to correct the changes in serum sodium and blood volume in consequence of disease severity [15]. These could be due to AVP-induced water retention and dilutional hyponatremia and hypokalemia.

In fact, our study has many limitations that should be addressed and clarified before getting the conclusion. First, the sample size of investigated COVID-19 patients, and being recruited from one medical center. Second, this was a single-point testing study without cohort follow-up and multiple sampling in a time progressive manner to correlate serum copeptin with the clinical outcomes. Third, the activation of the vasopressinergic system with subsequent increase in copeptin level is not exclusive or specific to COVID-19 cases only; however, the cut-off values and the pattern of increase could be promising for risk stratification. Fourth, atrial fibrillation was recently reported to be linked to COVID-19 infection, which may be a predictor for mortality but there was no data regarding atrial fibrillation for the patients in this study.

## Conclusion

Severe COVID-19 was associated with remarkable increase in serum copeptin level when compared to mild to moderate cases, with a reasonable sensitivity and specificity for differentiation. Therefore, copeptin may be a useful neuroendocrine biomarker for detecting severe COVID-19 cases on admission time to accelerate the intensive medical interventions for better life saving.

## Future perspective

Regarding the future perspective of our findings, it is expected with further studies on wider public samples that copeptin may have a role in the early classification of COVID-19 cases severity and prediction of the progression to facilitate early medical interventions and clinical decisions.

**Summary points**

- Copeptin is a neuroendocrine peptide mirroring the vasopressinergic system with great serum stability and easier detection.
- We hypothesized the implication of stress and COVID-19 severity in dysregulation of the vasopressinergic system with subsequent increase in serum copeptin level.
- Serum copeptin on admission time showed a significant increase in severe COVID-19 cases in comparison to the mild to moderate cases.
- Serum copeptin was positively correlated with CRP, ferritin and D-dimer, while it was negatively correlated with oxygen saturation, T-cells%, Na^+^, and K^+^.
- Among biochemical and inflammatory markers, at a serum level of 18.5 Pmol/l, copeptin showed the best diagnostic power for COVID-19 severity with sensitivity 93.33% and specificity 100%.
